# Pancreatic Cells Are Resistant to KRAS^Q61L^ Expression due to Hyperactive ERK/MAPK Signaling and Apoptosis Induction

**DOI:** 10.1158/2767-9764.CRC-25-0281

**Published:** 2025-10-22

**Authors:** Rachel A. Burge, Lucas Bialousow, Thomas McFall, Logan Bamonte, Grayson Johnson, Merissa Smith, Silvia G. Vaena, Susana Comte-Walters, Lauren E. Ball, Stefano Berto, John P. O’Bryan, G. Aaron Hobbs

**Affiliations:** 1 Department of Biochemistry & Molecular Biology, Medical University of South Carolina, Charleston, South Carolina.; 2 Department of Biochemistry and MCW Cancer Center, Medical College of Wisconsin, Milwaukee, Wisconsin.; 3 College of Graduate Studies, Medical University of South Carolina, Charleston, South Carolina.; 4 Bioinformatics Core, Medical University of South Carolina, Charleston, South Carolina.; 5 Department of Pharmacology and Immunology, Medical University of South Carolina, Charleston, South Carolina.; 6 Department of Neuroscience, Medical University of South Carolina, Charleston, South Carolina.; 7 Hollings Cancer Center, Medical University of South Carolina, Charleston, South Carolina.

## Abstract

The RAS family of small GTPases is among the most frequently mutated gene families in human cancer. In pancreatic ductal adenocarcinoma (PDAC), ∼95% of cases harbor an activating *KRAS* mutation, primarily at codon 12, 13, or 61, with G12D being the most common overall (40%). In contrast, the *KRAS*^*Q61L*^ mutation, though constitutively active, is virtually absent in tumors of patients with PDAC. This suggests that KRAS^Q61L^ may engage in distinct, allele-specific signaling that limits its ability to drive tumorigenesis. Determining the mechanisms that limit the occurrence of this mutation will aid in our understanding of the critical KRAS effectors and pathways that drive tumorigenesis. To investigate these mechanisms, we utilized a tightly controlled doxycycline-inducible KRAS expression system in an isogenic, immortalized pancreatic cell line, enabling direct comparison of KRAS^Q61L^ with the common PDAC mutant KRAS^G12D^. Using TurboID proximity labeling alongside RNA sequencing, we mapped early effector interactions and transcriptional responses, revealing that KRAS^Q61L^ induces greater hyperactivation of the ERK/MAPK pathway, resulting in increased nuclear translocation of ERK1/2. Finally, pancreatic cells are highly tolerant to overexpression of KRAS^G12D^, but KRAS^Q61L^ overexpression leads to impaired proliferation and increased apoptosis. These findings provide experimental support for the long-standing “Goldilocks” model of oncogenic signaling, in which too much ERK/MAPK pathway activation is detrimental to tumorigenesis. Our work offers a mechanistic explanation for the relative absence of KRAS^Q61L^ in PDAC and contributes to our understanding of KRAS allele–specific vulnerabilities, which can inform future therapeutic strategies targeting KRAS-driven pancreatic cancer.

**Significance:**

This study demonstrates that KRAS^Q61L^ drives hyperactivation of ERK/MAPK signaling and triggers apoptosis, which limits the proliferation of pancreatic cells. These findings support a “Goldilocks” model of RAS signaling and suggest that strong hyperactivation of the ERK/MAPK pathway contributes to the selective absence of KRAS^Q61L^ in pancreatic tumorigenesis.

## Introduction

Mutations in *RAS* genes are found in ∼30% of all human cancers, including a high prevalence in lung adenocarcinoma, colorectal cancer, and pancreatic ductal adenocarcinomas (PDAC), which are among the deadliest cancers in the United States (1–4). RAS is a small GTPase that acts as a molecular switch to regulate cell proliferation, survival, and differentiation. *KRAS* is the most frequently mutated RAS isoform (85%), followed by *NRAS* (12%) and *HRAS* [3%; Catalogue of Somatic Mutations in Cancer (COSMIC), version 102]. The majority of *RAS* mutations (∼98%) are single-nucleotide substitutions at three amino acid hotspots: glycine 12 (G12), glycine 13 (G13), and glutamine 61 (Q61). Mutations at codons G12, G13, and Q61 shift *RAS* to a constitutively active state and are broadly oncogenic (5, 6). *KRAS* mutations occur in ∼78% at G12, ∼14% at G13, and ∼3% at Q61 (COSMIC, version 102). The reason for this uneven mutational frequency in cancers is not well understood.

Differential environmental exposures related to the tissue of origin dictate the overall mutational spectrum in some cancers, such as lung cancer (7). Conversely, recent evidence indicates that the intrinsic biology of KRAS mutants results in selective effector interactions and altered downstream signaling, which play a role in shaping the mutational patterns observed in human cancers (8–14).

In PDAC, *KRAS* mutations are considered the initiating event and occur in ∼95% of cases. The most common *KRAS* mutations in PDAC are *KRAS*^*G12D*^ (42%), *KRAS*^*G12V*^ (31%), and *KRAS*^*G12R*^ (15%; GENIE cohort version 17.0). *KRAS* oncogenic hotspot mutations occur primarily at G12 and, at lower frequencies, at Q61 in pancreatic intraepithelial neoplasias (15). Interestingly, although the sample size was small, there was a higher frequency of Q61 mutations in pancreatic intraepithelial neoplasias (16%) than is typically seen in PDAC (2%). The rarity of KRAS^Q61^ mutations in PDAC suggests that hallmark oncogene characteristics, such as being GTP-locked and highly active, are not the only requirements for oncogenesis. A direct example of this is *KRAS*^Q61L^, which is rarely found in human cancers and accounts for less than 0.5% of all KRAS mutations in cancer. Previous work in NIH 3T3 and RIE cells has shown that *KRAS*^*Q61L*^ supports anchorage-independent growth (16), is constitutively GTP-locked, and strongly activates ERK1/2, all of which are hallmarks of oncogenic KRAS (9).

In colon and lung cancers, KRAS^Q61L^ mutations occur at a significantly higher frequency than predicted based on expected mutation frequency modeling (16), indicating that the biology of the tissue plays a role in the selection for specific KRAS mutants. Additionally, RAS^Q61x^ mutations were shown to be functionally distinct in their activation of the MAPK cascade (17). Combined, these observations support that not all KRAS mutants are equally capable of driving tumorigenesis and indicate that unique mutant-selective interactions may account for the low frequency of KRAS^Q61L^ mutations.

KRAS^Q61^ mutations impair intrinsic and GAP-mediated GTP hydrolysis more severely than codon 12 mutations, locking KRAS^Q61x^ in a GTP-bound state and leading to sustained RAF engagement and MAPK signaling (9). The “Goldilocks” model of RAS signaling suggests that although a threshold of pathway activation is necessary and dependent on hyperactivation of ERK signaling (18), excessive ERK signaling promotes senescence and apoptosis (19). The low frequency of KRAS^Q61x^ mutations in PDAC may reflect a signaling threshold beyond which ERK/MAPK pathway activation becomes detrimental.

Although many studies have focused on determining why mutations are enriched in specific cancers, we opted to take the opposite approach. In this study, we ask why KRAS^Q61L^, despite being highly oncogenic in model systems, is not routinely detected in pancreatic cancer. Utilizing protein proximity assays (BioID) in combination with poly(A) RNA sequencing (RNA-seq) and immunoblot analyses, we demonstrate that pancreatic cells are highly permissive to overexpression of KRAS^G12D^ but are not tolerant of KRAS^Q61L^ overexpression. Furthermore, we show that KRAS^Q61L^ hyperactivates the ERK/MAPK pathway to a greater degree than the more common oncogenic mutation KRAS^G12D^. This leads to enhanced nuclear translocation of ERK and greater upregulation of ERK transcriptional targets. We show that KRAS^Q61L^ is proteolytically degraded, which prevents elevated protein expression levels of this KRAS mutant. Despite the enhanced signaling, KRAS^Q61L^-harboring human ductal pancreatic nestin E6/E7 (HPNE) cells have slower apparent proliferation rates and increased apoptosis. Combined, these data support the “Goldilocks” hypothesis of RAS signaling (19) and provide a compelling mechanism for the rarity of KRAS^Q61L^ mutations in pancreatic cancer.

## Materials and Methods

### Cell lines and cell culture conditions

Primary mouse embryonic fibroblasts expressing only KRAS (DU1473; Hras^−/−^;Nras^−/−^;Kras^lox/lox^;RERT^ert/ert^) were generated and characterized previously (20) and were provided by the NCI RAS Initiative. Human telomerase reverse transcriptase–HPNE E6/E7 cell lines (RRMID:CVCL_C467) were created as previously described (21) and obtained from ATCC. All cell lines were maintained in high-glucose DMEM (Corning, #10-013-CV) supplemented with 10% FBS (VWR, #97068085) in a humidified chamber with 5% CO_2_ at 37°C without antibiotic treatment. HPNE KRAS-mutant cells were generated by stably expressing our HYGRO-pCW5.7 constructs encoding empty vector (EV), wild-type KRAS (KRAS^WT^), KRAS^G12D/R^, or KRAS^Q61L^. Lentivirus was produced by transfecting HEK293T cells (RRID:CVCL_0063) with the respective pCW5.7 construct along with packaging plasmids using FuGENE (RRID:SCR_016365), following the manufacturer’s protocol (Promega). Stable cell lines were maintained in medium supplemented with tetracycline-negative FBS (Gemini, #100-800-500). All media were filtered through a 0.22-micron filter prior to use. Stable cell lines were maintained in tetracycline-negative FBS (Gemini, #100-800-500). Unless otherwise indicated, the cells were not serum-starved prior to immunoblot analysis. The cell lines used in the experiments were passaged for approximately 10 to 15 passages before a new aliquot was thawed. Cell line authenticity was verified by short tandem repeat profiling of cell lines with available STR data, and all lines were monitored for mycoplasma contamination using the Lonza MycoAlert Mycoplasma Detection Kit.

### TurboID pull-downs

TurboID pull-downs were performed as previously described (22). Human pancreatic epithelial cells stably expressing HA-TurboID-KRAS fusion constructs were maintained in standard DMEM supplemented with 10% FBS. To reduce background biotinylation, cells were cultured in biotin-free media (dialyzed FBS) for 1 week prior to proximity labeling. For biotin labeling, cells were seeded in 10 cm dishes and grown to approximately 80% confluency. Media were replaced with fresh complete media containing 50 μmol/L biotin and incubated for 1 hour at 37°C. Following biotin labeling, cells were washed twice with PBS and lysed directly on plates using 540 μL/dish of freshly prepared lysis buffer (8 mol/L urea, 50 mmol/L Tris-HCl, pH 7.4, 1× EDTA-free protease inhibitor cocktail, and 1 mmol/L dithiothreitol). After scraping and transferring lysates to conical tubes, Triton X-100 was added to a final concentration of 1%. Lysates were sonicated using a Branson Sonifier 250 (30% duty cycle, output level 4) in two 60-pulse rounds with a 2-minute rest on ice between sessions. After dilution with 2.52 mL of cold lysis buffer, a third round of sonication (60 pulses) was performed. Lysates were aliquoted into 2-mL microcentrifuge tubes and centrifuged at 16,500 × *g* for 10 minutes at 4°C. Supernatants were transferred to pre-equilibrated Streptavidin Sepharose High Performance beads (GE Healthcare; 100 μL per sample) in fresh lysis buffer and incubated overnight at 4°C on a rotator. The next day, the bead-bound samples were pelleted by centrifugation (1,000 × *g*, 5 minutes), washed four times in urea-based wash buffer (8 mol/L urea, 50 mmol/L Tris-HCl, pH 7.4), and split for downstream applications. A 10% aliquot was resuspended in 1× SDS-PAGE sample buffer for Western blot analysis, whereas the remaining beads were resuspended in 50 μL of 50 mmol/L ammonium bicarbonate and flash-frozen in liquid nitrogen for mass spectrometry. Western blot samples were boiled at 98°C for 5 minutes and stored at −20°C until use.

### LC/MS-MS for proximity labeling BioID

Biotinylated proteins enriched with streptavidin beads were washed twice with 50 mmol/L ammonium bicarbonate (pH 8.5), reduced with 4.1 mmol/L dithiothreitol in 50 μL of 50 mmol/L ammonium bicarbonate, and cysteines were alkylated with 8.3 mmol/L iodoacetamide (Thermo Fisher Scientific). Proteins were digested off the beads with 200 ng of trypsin (Sigma-Aldrich, #650279) overnight at 37°C, and the resulting peptides were acidified, desalted using C18 Stage Tips (Thermo Fisher Scientific), and dried under vacuum. LC/MS-MS was performed on an EASY-nLC 1200 in line with the Orbitrap Lumos Mass Spectrometer (Thermo Fisher Scientific). Peptides were pressure loaded on an Acclaim PepMap RSLC, 75 μm × 50 cm (C18, 2 μm, 100 Å; Thermo Fisher Scientific) and chromatographically separated using a gradient of 5% to 35% B in 180 minutes (solvent A: 0.1% formic acid; solvent B: 80% acetonitrile and 0.1% formic acid) at 300 nL/minute and 45°C. Mass spectra were acquired in data-dependent mode with a high resolution (60,000) Fourier transform mass spectrometry survey scan, mass range of m/z 375 to 1,575, followed by collisional dissociation of the most intense precursors with a cycle time of 3 seconds. The automatic gain control target was 4 × 10^5^ for the survey mass spectrometry scan. The precursor isolation window was 1.6 m/z, the maximum injection time was 22 ms, and the higher-energy collisional dissociation collision energy was 35%. Tandem mass spectra were acquired in the Orbitrap at 15,000 resolution. Monoisotopic precursor selection was set to “peptide,” and “advanced peak determination” mode was enabled. Precursors were dynamically excluded from repeated sequencing for 25 seconds with a mass tolerance of ±10 ppm. Precursor ions with charge states that were undetermined, 1, or >7 were excluded. Data were searched against a reviewed human database (downloaded from UniProt on November 30, 2021) using MaxQuant v.2.0.1.0 (Max Planck Institute). The FDR for identification, determined using a decoy database strategy, was set at 1% at the protein and peptide level. Fully tryptic peptides with a minimum of seven residues were required. Parameters included static modification of cysteine with carbamidomethyl, variable N-terminal protein acetylation, and oxidation of methionine. Two missed cleavages were permitted. A 4.5 ppm tolerance was used for the main search. Matching between runs was enabled. A minimum label-free quantitative (LFQ) ratio count of 2 was required for protein quantification, with at least one unique peptide. The MaxQuant protein groups’ text file was processed in Perseus version 1.6.15.0 (Max Planck Institute). Proteins identified by a single peptide, matches to the decoy database, and common contaminants were removed. The normalized, log_2_-transformed LFQ protein intensities were filtered to retain proteins quantified in at least three biological replicates in one group. Missing values were imputed from a normal distribution with a width of 0.3, downshifted by 1.8. Protein intensities were compared using a Student *t* test.

### Streptavidin blotting

To confirm TurboID-dependent biotinylation, SDS-PAGE–resolved proteins were transferred to nitrocellulose membranes and processed using streptavidin conjugates. Membranes were washed in TBS + 0.1% Tween-20 (TBST), blocked in 5% nonfat dry milk in TBS, and incubated for 1 hour at room temperature with streptavidin–HRP (1:5,000) in TBST + 1% milk. Following antibody incubation, membranes were washed sequentially in TBST, TBS, and sterile water and developed using standard chemiluminescent detection methods.

### Generation of protease-resistant streptavidin beads

To enhance compatibility with downstream proteomic workflows, streptavidin beads were rendered trypsin-resistant via two-step chemical modification, as published previously (23). Briefly, 250 μL of beads were washed in PBS + 0.1% Tween-20 (PBST) and incubated in 700 μL of 1,2-cyclohexanedione in PBST (pH 13) for 4 hours at room temperature. Beads were washed and subsequently incubated in 700 μL of 4% formaldehyde and 700 μL of 0.2 mol/L sodium cyanoborohydride for 2 hours. Beads were washed sequentially with 0.1 mol/L Tris-HCl (pH 7.5) and PBST and stored in 250 μL of PBST at 4°C.

### Immunoblotting

Cells were washed twice with ice-cold PBS, lysed in 1% NP-40 buffer [25 mmol/L HEPES (pH 7.4), 100 mmol/L NaCl, 10 mmol/L MgCl_2_, 1% NP-40, 1% glycerol, and 0.1% SDS] supplemented with phosphatase I (MilliporeSigma, #524624), phosphatase II (MilliporeSigma, #P5726), and protease inhibitors (MilliporeSigma, Roche, #11697498001); scraped; and collected in chilled Eppendorf tubes. Lysates were cleared by centrifugation at 18,000 × *g* for 20 minutes at 4°C, and protein concentration was determined using the Bradford assay (Bio-Rad, #5000006). Immunoblotting was performed using a standard protocol. The membranes were blocked with 5% milk diluted in TBST. To determine the levels of activated proteins, Western blot analyses were performed using the following antibodies: KRAS (RRID:AB_1842235), AKT (RRID:AB_329827), pAKT (S473; RRID:AB_2315049), pAKT (T308; RRID:AB_2255933), vinculin (RRID:AB_2728768), pERK1/2 (RRID:AB_2315112), ERK1/2 (RRID:AB_330744), HA-tag (RRID:AB_1549585), cleaved caspase-3 (RRID:AB_2070042), and caspase-3 (RRID:AB_2070042).

### RNA-seq

Sequencing libraries for the HPNE cell lines were prepared at the Medical University of South Carolina Translational Science Lab. Briefly, polyadenylated RNA was extracted from 1 μg of total RNA per sample, and libraries were prepared using the NEBNext Poly(A) mRNA Magnetic Isolation Module and the NEBNext Ultra II Directional RNA Library Prep Kit for Illumina (New England Biolabs, #E7760L). Paired-end sequencing was performed at the Vanderbilt VANTAGE core laboratory (Vanderbilt University) to a depth of 25 million reads per library using an Illumina NovaSeq 6000 (RRID:SCR_020150). Sequencing data were analyzed using Partek Flow software. Reads were aligned to the human genome assembly hg38 using spliced transcript alignment to a reference (STAR, RRID:SCR_004463) and quantified using an annotation model (Partek E/M, RRID:SCR_011860) using hg38. The reads were normalized using the Trimmed Mean of M components and gene set enrichment analysis (GSEA) using the Human Collection Molecular Signatures Database Human Hallmarks version 2024 (RRID:SCR_003199). Heatmaps were created by taking significant normalized enrichment scores from GSEA.

### Bioluminescence resonance energy transfer assays

Human embryonic kidney HEK293T cells were grown in DMEM/10% FBS without antibiotic treatment. Cells were seeded at a density of 5,000 cells per well in a 96-well white opaque PerkinElmer microplate. Twenty-four hours after seeding, cells were cotransfected with a constant concentration of 0.1 μg CRAF-NanoLuc pcDNA expression plasmid and increasing concentrations of GFP-tagged KRAS mutant with 0.25 μL of Lipofectamine 3000 per well following the manufacturer’s protocol (Thermo Fisher Scientific, #L3000001). Twenty-four hours later, the medium was aspirated from each well, and 25 μL of Nano-Glo Live Cell Reagent was added to each well according to the manufacturer’s protocol (Promega, # N2011). The plates were then placed on an orbital shaker for 1 minute at 30 rpm. After incubation, the plate was read on a Tecan Infinite M200 PRO with Lumicolor Dual Setting, with an integration time of 1,000 ms. The bioluminescence resonance energy transfer (BRET) ratio was calculated from the dual emission readings. The BRET ratio was plotted as a function of the RAS-GFP/NF1-NanoLuc ratio. The BRET assays were repeated 3 times, each with eight biological replicates. All raw BRET ratios were plotted along with luciferase and GFP units (read before the addition of substrate to the wells).

### Proliferation assays

To measure proliferation on plastic, cells were plated in technical replicates in 12-well dishes at a density of 2 × 10^3^. Plates were developed after 7 days by removing the medium and fixing cells with 4% paraformaldehyde and crystal violet. Each biological replicate experiment included three technical repeats for each cell line.

### Cellular thermal stability assays

Cellular thermal stability assay (CETSA) was performed to assess the thermal stabilization of KRAS mutants in intact cells. KRAS^G12D/Q61L^-HPNE cells were seeded and grown to ∼80% confluence. Cells were then trypsinized, and one million cells were collected by centrifugation, washed with cold PBS, and resuspended in PBS supplemented with protease inhibitors. Aliquots were subjected to a thermal gradient ranging from 45°C to 85°C (in 5°C increments) for 3 minutes using a thermal cycler, followed by a 3-minute incubation at room temperature. Cells were lysed by three freeze–thaw cycles using liquid nitrogen and a 25°C water bath. Lysates were centrifuged at 20,000 × *g* for 20 minutes at 4°C to separate soluble proteins from aggregated material. Supernatants were collected and analyzed by SDS-PAGE and Western blotting using an anti-KRAS antibody. Band intensities were quantified and plotted to generate melting curves.

### Statistical analysis

Data were analyzed using GraphPad Prism (RRID:SCR_002798) built-in tests (one-way ANOVA, Dunnett’s multiple comparisons test) or Student *t* test in R 4.2.2. Data are presented relative to the respective controls or as noted in the figure legend. Error bars indicate mean ± SEM for ≥3 independent experiments (except where noted), and *P* values on graphs are denoted by ****, *P* < 0.0001; ***, *P* < 0.0005; **, *P* < 0.002; and *, *P* < 0.05 or as indicated in each figure legend. The number of samples analyzed per experiment and whether the data are representative or averaged are indicated in the figure legend. Group size determination and power calculations were not applicable, as this study was conducted exclusively in cell lines without the use of animal or human subjects. Blinding of investigators was not performed, as quantitative analysis using Western blot and RNA-seq minimized the potential for bias in data interpretation. Sex was not considered a relevant biological variable in this study, as experiments were conducted in established pancreatic epithelial cell lines under controlled *in vitro* conditions, in which sex-specific influences are minimized or not preserved.

## Results

### KRAS^Q61L^ hyperactivates ERK1/2 while limiting proliferation in pancreatic cancer cells

KRAS mutations are considered the initiating event in PDAC and are thought to drive early neoplastic transformation (24–26). Despite being highly transforming in biochemical studies (16), KRAS^Q61L^ mutations are rare in PDAC. We predict that understanding what makes a mutation rare will aid in our ability to better target KRAS signaling in oncogenic settings.

**Figure 1.**
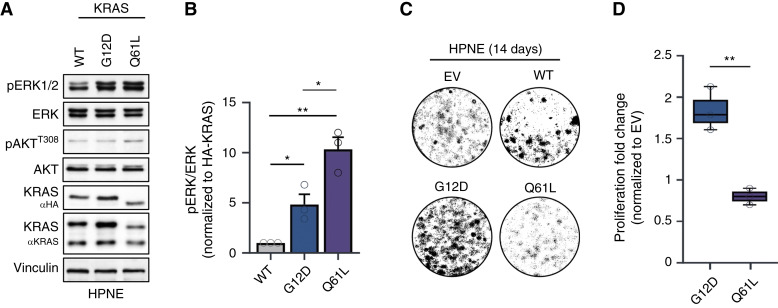
KRAS^Q61L^ hyperactivates ERK1/2 while limiting proliferation in pancreatic cancer cells. **A,** Immunoblot of KRAS mutants expressed in human telomerase reverse transcriptase–HPNE E6/E7 cell lines for 48 hours with DOX-induced KRAS expression (*n* = 3). **B,** Quantification of pERK1/2 in HPNE KRAS-mutant cell lines from three independent experiments. **C,** Representative images of KRAS^WT/G12D/Q61L^ and EV expressed in human telomerase reverse transcriptase–HPNE E6/E7 cell lines (*N* = 3). **D,** Box and whisker plot quantifying the proliferation of HPNE cells expressing KRAS^G12D/Q61L^ from **C**. KRAS mutants were normalized to HPNE EV. *P* values were calculated using the Student *t* test. Error bars represent mean ± SEM. **, *P* < 0.002; *, *P* < 0.05. All statistical tests were run using R 4.2.2.

To examine signaling and proliferation of KRAS^Q61L^ in the context of pancreatic cells, we ectopically expressed KRAS^G12D^ and KRAS^Q61L^ in human telomerase reverse transcriptase–HPNE cells. HPNE cells were previously immortalized by ectopic telomerase expression and have TRP53 inactivated by the stable transduction of E6/E7 (21). In this study, KRAS-mutant protein expression was controlled via doxycycline (DOX) to modulate ectopic expression of the mutant KRAS proteins. To explore early KRAS-driven perturbations to the cell, we cultured cells in tetracycline-free media and treated them with DOX for 48 hours before analysis. Compared with KRAS^G12D^, KRAS^Q61L^ hyperactivated the ERK1/2 MAPK pathway with lower levels of protein expression (Fig. 1A and B). Curiously, KRAS^Q61L^ seemed resistant to overexpression and required more DOX to obtain similar levels of ectopic protein expression. To rule out variations in viral transduction, the cell lines were generated multiple independent times, and KRAS^Q61L^ consistently displayed reduced protein expression levels, whether probed using anti-KRAS or anti-HA antibodies. In line with previously published reports (9, 16), a KRAS GTP pull-down of cells harboring KRAS^G12D^ and KRAS^Q61L^ revealed KRAS^Q61L^ to have increased GTP loading compared with KRAS^G12D^ (Supplementary Fig. S1A and S1B). KRAS^Q61L^-expressing HPNE cells had significantly slower proliferation rates compared with KRAS^G12D^ (Fig. 1C and D). Thus, these data suggest that hyperactivation of the ERK pathway does not in and of itself lead to increased proliferation but is context-dependent.

### BioID protein proximity labeling detected similar protein interactomes

**Figure 2.**
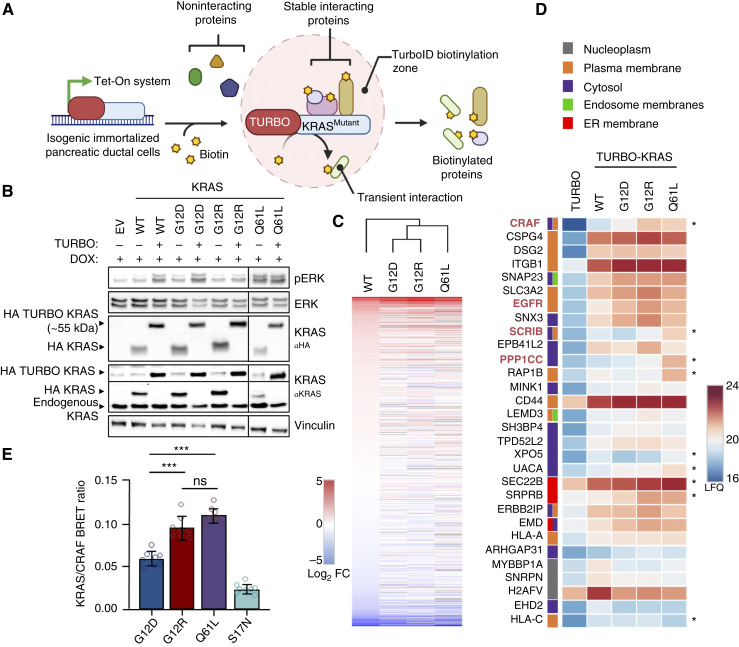
KRAS^Q61L^ has increased localization with CRAF effectors and higher CRAF affinity in comparison with KRAS^G12D^. **A,** Schematic of TURBO attached to KRAS mutants expressed in human telomerase reverse transcriptase–HPNE E6/E7 cell lines. **B,** Immunoblot of KRAS and TURBO-KRAS mutants expressed in human telomerase reverse transcriptase–HPNE E6/E7 cell lines for 48 hours with DOX-induced expression (*N* = 3). **C,** Heatmap of log_2_ fold change of TURBO-KRAS in comparison with TURBO alone and ranked based on KRAS^WT^. **D,** Heatmap showing the relative abundance (LFQ intensities) of the top 25 interactors of both KRAS^G12D^ and KRAS^Q61L^ mutants compared with TURBO alone, based on mass spectrometry analysis. Subcellular fractionation was assessed using Reactome. **E,** Quantification of BRET measurements for CRAF with KRAS^G12D/R/Q61L^ and KRAS^S17N^. Data are the average from three independent experiments with eight technical replicates. *P* values were calculated using one-way ANOVA. Error bars represent mean ± SEM. All statistical tests were run using R 4.2.2. ***, *P* < 0.0005; *, *P* < 0.05; ns, not significant; FC, fold change. [**A** and **D,** created in BioRender. Burge, R. (2025) https://BioRender.com/g53ttip.]

As KRAS^Q61L^ and KRAS^G12D^ activate the ERK/MAPK pathway but differ in their ability to promote HPNE cell proliferation, we performed an unbiased protein proximity assay to assess mutant-selective protein interactions. We compared KRAS^Q61L^ with two common PDAC mutations: KRAS^G12D^ and KRAS^G12R^, utilizing BioID (27) in HPNE cells (Fig. 2A). We first assessed whether the TurboID accessory protein altered the ability of the KRAS mutants to promote ERK activation. As expected, the addition of TurboID to the N-terminus of KRAS mutants had no effect on their ability to promote ERK/MAPK activation or alter morphology (Fig. 2B; Supplementary Fig. S2A).

Previous biotin proximity labeling assays have been used to determine mutant RAS interactomes (28–30). However, these approaches did not express near-endogenous levels of the labeled KRAS protein in a pancreatic cell environment. We utilized a DOX-inducible system to regulate protein expression of TURBO-KRAS to levels that were similar to endogenous KRAS levels to minimize overexpression artifacts and ensure we were detecting biologically relevant mutant-selective interactions. Biotinylation of proteins in each cell line was performed in biological triplicate, and we confirmed that protein biotinylation was due to the addition of TurboID in the presence of biotin (Supplementary Fig. S2B and S2C). Biotinylated proteins were affinity purified with streptavidin agarose and identified using LC/MS-MS to generate LFQ protein intensities (Supplementary Fig. S2D; Supplementary Table S1). When comparing the TURBO-KRAS mutants, we observed highly similar interactomes, but hierarchical clustering placed KRAS^G12D^ and KRAS^Q61L^ furthest apart, suggesting the greatest differences in their interaction networks (Fig. 2C).

### KRAS^Q61L^ has increased association with ERK/MAPK pathway proteins in HPNE cells

Many of the top hits correlated with the expected GTP/GDP ratio: WT < G12D < G12R < Q61L. We compiled the top 25 interactors enriched with KRAS^Q61L^ compared with TurboID and the top 25 enriched with KRAS^G12D^, resulting in a combined list of 30 proteins, as the majority of top interactors were shared between the two mutants. Nine proteins in this heatmap have an increase of ≥ ±0.5 log_2_ fold change between KRAS^Q61L^ and KRAS^G12D^ (asterisks; Fig. 2D). To further contextualize these interactions, we classified proteins based on their predominant subcellular localization. Within the top 25 hits for each mutant, proteins such as CRAF, EGFR, PPP1CC (PP1C), and SCRIB are directly associated with ERK/MAPK signaling (highlighted in red), whereas others, including ITGB1 and CD44, are associated with the plasma membrane (orange box). Most interactions across cellular compartments were relatively similar between KRAS variants. Endoplasmic reticulum–associated proteins (red box) may reflect expression-related localization, whereas cytosolic proteins (purple box) may represent trafficking machinery or proteins situated near membrane surfaces.

We directly compared KRAS^G12D/R^ with KRAS^Q61L^ and generated heatmaps to visualize changes in protein interactions (Supplementary Fig. S2E and S2F). We removed proteins that showed strong LFQ intensity in the TurboID-alone control to eliminate background interactions. The most significant interaction hits enriched in KRAS^Q61L^ included SCRIB, RAP1B, and PPP1CC. These results suggest that although KRAS^Q61L^ and KRAS^G12D^ engage similar protein networks, KRAS^Q61L^ exhibits generally stronger interactions with proteins associated with the ERK/MAPK pathway. This conclusion aligns with our immunoblot analyses showing that KRAS^Q61L^, although having lower levels of expression, robustly activates ERK/MAPK signaling.

There were some notable exceptions. The nuclear export–related protein XPO5 was enriched in KRAS^Q61L^, and XPO5 specializes in RNA transport from the nucleus to the cytoplasm. RAP1B was also significantly higher in the KRAS^Q61L^ TurboID data, and RAP1B is found at the plasma membrane, in which it promotes several different cellular functions. Critically, RAP1B associates with BRAF and has been shown to enhance KRAS-MAPK signaling by coactivating BRAF (31) although it remains unclear whether this interaction promotes or modulates signaling through competitive binding. SEC22B and SRPRB are both involved in subcellular trafficking, and together, these interactions could reflect altered compartmentalized signaling or differences in protein trafficking efficiency, folding, or modification (32, 33). UACA, or “nucling,” a protein shown to promote stress-induced apoptosis (34), had increased localization with KRAS^Q61L^. Overall, the KRAS^Q61L^ interactome was most enriched for proteins involved in MAPK/ERK regulation. These data suggest that KRAS^Q61L^ engages a largely overlapping set of interactors as KRAS^G12D^ but exhibits increased association with ERK/MAPK pathway components.

To further investigate the possibility that KRAS^Q61L^ favors enhanced MAPK pathway activation, we assessed whether KRAS^Q61L^ has a higher affinity for CRAF. Using in-cell BRET analysis, we show that KRAS^Q61L^ has a significantly greater interaction with CRAF compared with KRAS^G12D^ (Fig. 2E), further supporting our conclusion that KRAS^Q61L^ has increased dwell time with regulators of ERK/MAPK. Previously, this increased BRET signal has been associated with an increase in the relative fraction of GTP loading of KRAS^Q61L^ relative to KRAS^G12D^ (35, 36) and is likely the significant contributing factor in our analysis as well. In agreement, our previous studies did not detect a difference in absolute binding affinity between KRAS^Q61L^ and KRAS^G12D^ (16) or KRAS^G12R^ and KRAS^G12D^ (10).

Finally, detection via the BioID protein proximity assay does not guarantee direct protein interactions. We attempted to validate SCRIB/PP1C interactions with KRAS^Q61L^ using immunoprecipitation but were unable to detect a stable interaction (Supplementary Fig. S2G and S2H). These observations suggest that the mutations do not differ in effector binding but rather in the strength or temporal extent of those interactions.

### KRAS^Q61L^ has a stronger PDAC-KRAS transcriptional signature than KRAS^G12D^

**Figure 3.**
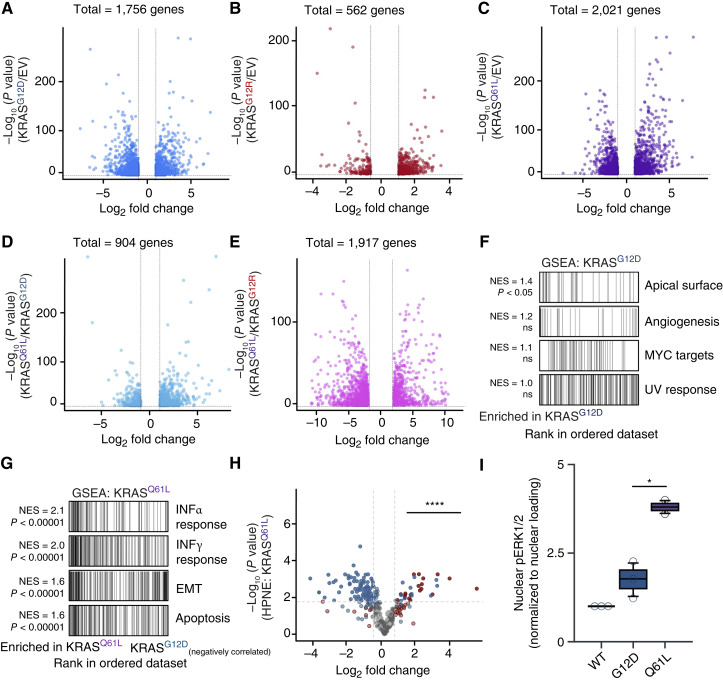
The rare KRAS^Q61L^ mutation drives a stronger PDAC-KRAS transcriptional signature than two common PDAC mutations. **A,** Significant genes in KRAS^G12D^ compared with EV. **B,** Significant genes in KRAS^G12R^ compared with EV. **C,** Significant genes in KRAS^Q61L^ compared with EV. **D,** Significant genes in KRAS^Q61L^ compared with KRAS^G12D^. **E,** Significant genes in KRAS^Q61L^ compared with KRAS^G12D^. **F,** Volcano plot with changes in the 200 KRAS-dependent (upregulated, red) and KRAS-inhibited (downregulated, blue) genes in the PKS in KRAS^Q61L^ compared with EV. All nonsignificant changes are denoted in gray (adjusted *P* > 0.05), and lighter colors indicate nonsignificant <0.5 log_2_ fold change. The dotted lines represent the cutoff of 0.05 adjusted *P* value and ± 0.5 log_2_ fold change. **G,** Significant hallmark pathways in HPNE KRAS^G12D^ compared with KRAS^Q61L^ using GSEA hallmark analysis. Normalized enrichment scores (NES) and adjusted *P* values are listed for all corresponding pathways. **H,** Significant hallmark pathways in HPNE KRAS^Q61L^ compared with HPNE KRAS^G12D^ using GSEA hallmark analysis. EMT: epithelial–mesenchymal transition. NES and adjusted *P* values are listed for all corresponding pathways. **I,** Box and whisker plot quantifying nuclear pERK1/2 levels from HPNE subcellular fractionation. pERK1/2 was normalized to the loading control in the nuclear fraction. *P* values were calculated using the Student *t* test. Error bars represent mean ± SEM. ****, *P* < 0.0001; *, *P* < 0.05. All statistical tests were run using R 4.2.2.

We next compared the transcriptional signature of KRAS mutants expressed in HPNE cells for 48 hours. We performed poly(A) RNA-seq in HPNE cells expressing an EV, KRAS^G12D/R^, or KRAS^Q61L^. After 48 hours of KRAS^G12D^ expression, we detected significant changes in 1,756 genes in comparison with the EV control (Fig. 3A). In line with previous reports of KRAS^G12R^-mutant PDAC being weaker than KRAS^G12D^-PDAC (37), we detected a much lower number of significantly altered genes in KRAS^G12R^ (Fig. 3B). In KRAS^Q61L^-expressing cells, we detected an increase/decrease in 2,021 genes when compared with the EV control (Fig. 3C). We then compared KRAS^Q61L^ directly with KRAS^G12D^ and detected ∼900 differentially expressed genes (Fig. 3D). Comparing KRAS^Q61L^ with KRAS^G12R^ showed an even greater number of differentially regulated genes (1,917; Fig. 3E). This comparison highlights the stronger transcriptional impact due to KRAS^Q61L^ expression (Fig. 3D and E). To visualize the overlap and differences in gene expression changes among these analyses, we generated Venn diagrams highlighting the significantly altered genes for each pairwise comparison (Supplementary Fig. S3A and S3B).

We next performed GSEA to compare transcriptomic differences between KRAS^G12D^ and KRAS^Q61L^ in HPNE cells. KRAS^G12D^ expression, compared with KRAS^Q61L^, showed an enrichment in only one pathway, the apical surface, with a modest normalized enrichment score of 1.4 (Fig. 3F). In contrast, KRAS^Q61L^ expression, when compared with KRAS^G12D^, was enriched in 15 pathways, including strong activation of interferon responses, epithelial–mesenchymal transition, and apoptosis (Fig. 3G). Importantly, most pathways significantly enriched in KRAS^Q61L^ compared with KRAS^G12D^ were also significantly enriched in both KRAS mutants when compared with EV (Supplementary Fig. S3C). This result suggests that KRAS^Q61L^ drives similar transcriptional programs as KRAS^G12D^ but with greater magnitude. Notably, the “KRAS signaling UP” and “apoptosis” pathways were negatively enriched in KRAS^G12D^ compared with the EV control, whereas both were positively enriched in KRAS^Q61L^ relative to EV. Overall, these findings suggest that KRAS^Q61L^ more potently upregulates mutant KRAS transcriptional programs early after expression.

Recently, the PDAC-specific KRAS transcriptional signature (PKS) was published, which suggested that the dominant role of mutant KRAS in PDAC was to drive activation of the RAF/MEK/ERK pathway (38, 39). As our BioID data indicated that KRAS^Q61L^ largely interacted with only the RAF/MEK/ERK signaling pathway, we questioned whether this interaction resulted in an altered transcriptional output. To investigate how MAPK pathway activity influences gene expression downstream of KRAS^Q61L^, we took 200 upregulated KRAS-dependent genes (colored red: PDAC “UP”) and 200 downregulated KRAS-dependent genes (colored blue: PDAC “DN”) from the PKS and compared the expression profiles of KRAS^G12D^ and KRAS^Q61L^. PKS analysis revealed robust activation of KRAS-driven transcription in KRAS^Q61L^-expressing HPNE cells. Although both mutants showed similar downregulation of PDAC “DN” genes, KRAS^Q61L^ induced significantly higher transcriptional expression of PDAC “UP” genes than both KRAS^G12R^ and KRAS^G12D^ (Fig. 3H; Supplementary Fig. S3D and S3E).

Given that KRAS^Q61L^ most robustly upregulated KRAS transcriptional networks, we questioned whether this was due to phosphorylated ERK translocating to the nucleus more efficiently or via some other mechanism. As phosphorylated ERK only elicits transcriptional changes when in the nucleus (40), we examined the nuclear fraction from HPNE cells. In agreement, KRAS^Q61L^ triggered higher levels of nuclear pERK1/2 than KRAS^G12D^ even though KRAS^Q61L^ protein levels were substantially lower (Fig. 3I; Supplementary Fig. S3F). This suggests that even with reduced total protein expression, KRAS^Q61L^ is highly efficient at driving ERK/MAPK signaling. Thus, in cells of pancreatic origin, KRAS^Q61L^ drives stronger ERK/MAPK pathway activation compared with both KRAS^G12D^ and KRAS^G12R^, which results in elevated levels of nuclear pERK1/2 and increased transcriptional output from activated ERK.

### KRAS^Q61L^ is detrimental to cell proliferation

**Figure 4.**
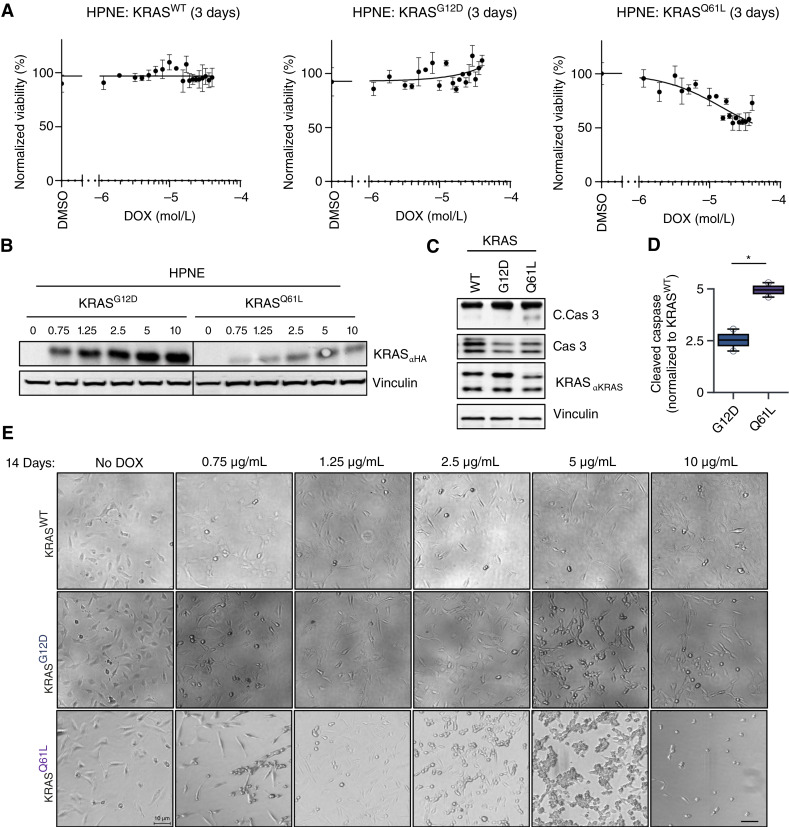
KRAS^Q61L^ expression limits proliferation due to induction of apoptosis. **A,** Cell proliferation assays with KRAS mutants expressed in human telomerase reverse transcriptase–HPNE E6/E7 cell lines for 72 hours. Cells were treated with increasing concentrations of DOX to drive mutant KRAS expression. Each condition includes three technical replicates per biological replicate; data shown are representative of two independent experiments. **B,** Immunoblot of DOX titration of KRAS^G12D^ and KRAS^Q61L^ in HPNE cells (*N* = 3). **C,** Immunoblot analysis of KRAS mutant–expressing cells following DOX induction, probing for cleaved caspase-3 (C.Cas-3) and caspase-3 (Cas-3) to assess apoptosis. **D,** Box and whisker plot quantifying the C.Cas-3 to Cas-3 ratio, normalized to KRAS expression, from **C** (*N* = 2). **E,** Representative bright-field images of HPNE KRAS^WT/G12D^ and KRAS^Q61L^ cells after long-term DOX induction (14 days), showing morphologic changes. *P* values were calculated using the Student *t* test. Error bars represent mean ± SEM. *, *P* < 0.05. All statistical tests were run using R 4.2.2. [**D,** created in BioRender. Burge, R. (2025) https://BioRender.com/xer49di.]

Given that KRAS^Q61L^ strongly activates the ERK/MAPK pathway and prior studies in skin cancer and leukemia have shown that excessive MAPK signaling can be detrimental (19, 41, 42), we investigated the relationship between ERK activity and cell proliferation. Because KRAS^G12R^ has already been extensively compared with KRAS^G12D^ (10, 37), we focused our analysis on KRAS^G12D^ and KRAS^Q61L^. We hypothesized that proliferation in HPNE cells is driven more by KRAS-driven ERK activity than by mutation-specific signaling differences (9, 10, 14, 37, 43–45). To test this, we titrated DOX concentrations to modulate KRAS expression and measured both KRAS protein levels and short-term cell proliferation (3 days). DOX addition had no effect on proliferation in HPNE cells harboring ectopic KRAS^WT^. Increasing DOX concentrations led to higher KRAS^G12D^ expression and modestly higher proliferation in KRAS^G12D^-expressing HPNE cells after 3 days (Fig. 4A and B). In contrast, KRAS^Q61L^-expressing cells failed to increase mutant protein levels with higher DOX concentrations (Supplementary Fig. S4), and cell proliferation rapidly trended negative (Fig. 4A and B). To align with the transcriptional increase in apoptosis-related genes, we probed for cleaved caspase-3 via immunoblot, which confirmed that reduced proliferation in KRAS^Q61L^-expressing cells was due in part to increased apoptosis (Fig. 4C and D). Additionally, bright-field imaging after 14 days of DOX treatment revealed pronounced cell rounding in KRAS^Q61L^-expressing HPNE cells at higher DOX doses, consistent with apoptosis (Fig. 4E). Treatment with similar DOX concentrations in KRAS^WT^ and KRAS^G12D^ cells indicated that this phenotype was not due to the addition of DOX alone. These findings support that elevated KRAS^Q61L^ expression induces apoptosis in pancreatic cells through ERK/MAPK hyperactivation.

### KRAS^Q61L^ is trafficked to the proteasome

**Figure 5.**
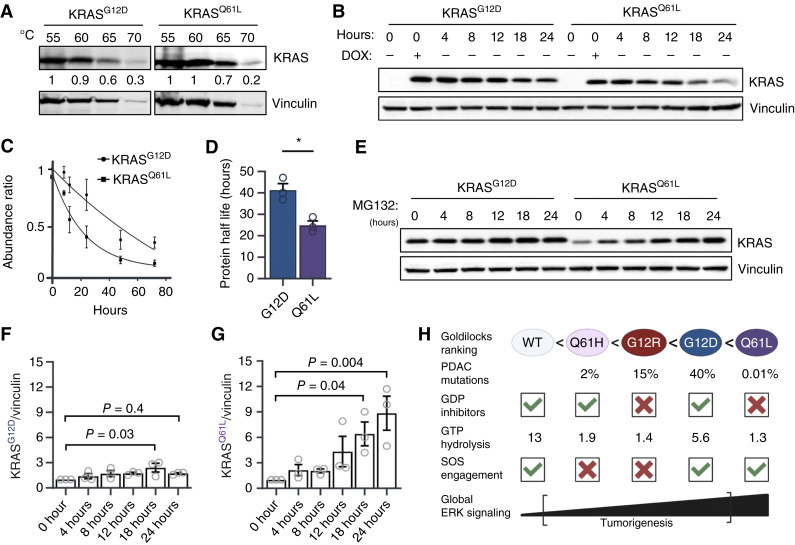
Increased proteasomal and membrane-associated degradation limits KRAS^Q61L^ expression in pancreatic cells. **A,** Thermostability of KRAS^G12D^ and KRAS^Q61L^ assessed using whole-cell lysates from HPNE cells subjected to increasing temperatures, followed by centrifugation to separate soluble and insoluble fractions. Immunoblots were used to compare the temperature at which the majority of KRAS becomes insoluble. Quantification of KRAS-mutant protein levels at each temperature is shown below the KRAS total blot. **B,** Immunoblot of KRAS^G12D^ and KRAS^Q61L^ protein levels in HPNE cells following DOX withdrawal. A representative image of three independent experiments is shown. **C,** KRAS^G12D^ and KRAS^Q61L^ protein half-lives using analysis of immunoblots from **B**. Protein decay curves were fit to a one-phase exponential decay model using the following equation: Y = Y_0_ × e^(−kt)^, where Y is the amount of protein at time t, Y_0_ is the starting amount of protein, k is the decay rate constant, and t is time. Protein half-lives (t_1_/_2_) were calculated using the following formula: t_1_/_2_ = ln(2)/k. **D,** Quantification of KRAS^G12D^ and KRAS^Q61L^ protein half-lives using analysis of protein decay curves from **C**. **E,** Immunoblot analysis of KRAS^G12D^ and KRAS^Q61L^ following treatment with the proteasome inhibitor MG132 over the indicated time course in HPNE cells. **F,** Quantification of KRAS^G12D^ from **E**. **G,** Quantification of KRAS^Q61L^ from **E**. **H,** Schematic of KRAS mutations and hypothesized “Goldilocks” ranking based on inhibitor sensitivity, GTP hydrolysis rates, and SOS sensitivity. *P* values were calculated using a Student *t* test or a one-way ANOVA. *, *P* < 0.05 or as indicated. Error bars represent mean ± SEM. Statistical tests were performed using R version 4.2.2. [Graphs were created in PRISM, and **H,** created in BioRender. Burge, R. (2025) https://BioRender.com/9phro2jschematics].

As KRAS^Q61L^ protein levels were unable to be overexpressed to similar levels as KRAS^G12D^, we hypothesized that intracellular mechanisms were restricting KRAS^Q61L^ expression. Foremost, we asked whether KRAS^Q61L^ was less thermodynamically stable than KRAS^G12D^. Previous studies using bacterially generated mutant KRAS proteins had not detected significant mutant-specific differences between KRAS^G12D^ and KRAS^Q61L^ in protein thermal stability (46). However, bacterially produced proteins do not contain a fully processed C-terminus, and it remains possible that these regions may differentially interact with the G domain of mutant RAS. Therefore, we performed the CETSA (47) to measure intracellular protein stability (Fig. 5A). Although CETSA was originally pioneered to detect changes in protein stability due to small-molecule binding, we rationalized that we could perform this assay with different KRAS mutants and monitor overall protein stability. By quantifying the amount of remaining soluble protein at each temperature, we determined the melting temperature (Tm), at which 50% of the protein remains soluble. We measured a Tm for KRAS^G12D^ of approximately 65°C, in line with previous studies (48). Surprisingly, KRAS^Q61L^, while less overall total protein was detected, also displayed a Tm of approximately the same temperature. Thus, thermostability does not explain the inability to increase KRAS^Q61L^ protein levels in HPNE cells.

We then investigated whether differences in translational processing contributed to the differential protein expression levels observed between the two KRAS mutants. To assess this, we performed a DOX washout experiment and monitored KRAS protein expression over time, effectively serving as a protein chase assay. As our constructs are DOX-inducible, DOX washout should lead to comparable degradation of mRNA transcripts across mutants, allowing differences in protein levels to reflect differences in protein turnover and/or stability. In this assay, KRAS^Q61L^ protein levels declined more rapidly than KRAS^G12D^ over 24 hours after DOX removal (Fig. 5B). KRAS^Q61L^ has a significantly shorter protein half-life compared with KRAS^G12D^ (Fig. 5C and D), suggesting that increased protein degradation/turnover contributes to the limited accumulation of KRAS^Q61L^.

Finally, we investigated whether KRAS^Q61L^ is degraded via the proteasome, which is responsible for the majority of protein turnover in mammalian cells (35). Inhibition of the proteasome with MG132 rescued the otherwise low expression levels of KRAS^Q61L^, indicating that this mutant is subject to active proteolytic degradation (Fig. 5E). In contrast, MG132 had a minimal effect on KRAS^G12D^ protein levels, further suggesting that KRAS^Q61L^ is selectively targeted for proteasomal degradation (Fig. 5F and G). After MG132 treatment, whole-cell lysates were collected and immunoblotted for the HA epitope tag. Treatment with MG132 for 24 hours resulted in a ladder of higher molecular weight species above the unmodified HA-KRAS band. These higher molecular weight bands were assumed to be ubiquitinated HA-tagged KRAS. KRAS^G12D^ and KRAS^Q61L^ accumulated similar levels of higher molecular weight HA-KRAS species, and both mutants were lower than WT, suggesting that direct ubiquitin-mediated degradation is not the primary mechanism regulating KRAS^Q61L^ stability when overexpressed (Supplementary Fig. S5A). Gene ontology analysis revealed significant enrichment of protein proteolysis–related processes in KRAS^Q61L^-expressing cells compared with KRAS^G12D^ (Supplementary Fig. S5B). Recent studies have shown that ubiquitin-dependent and ubiquitin-independent mechanisms can mediate KRAS proteasomal degradation (36, 37). Thus, further work needs to be performed to elucidate the specific pathway(s) responsible for the increased KRAS^Q61L^ protein turnover.

To extend our analysis beyond the pancreas, we sought to explore KRAS^Q61L^ mutations in other cancer types using publicly available RNA sequencing databases. However, KRAS^Q61x^ mutations are exceedingly rare, accounting for only 2.5% of all KRAS hotspot mutations, which is strikingly low compared with their prevalence in NRAS (31%) and HRAS (COSMIC; Supplementary Fig. S5C; ref. 35). We probed multiple publicly available cancer genomic databases for RNA sequencing data. However, with such low sample numbers, the feasibility of a meaningful cross-cancer analysis was not possible (Supplementary Fig. S5D). The overall rarity of the KRAS^Q61L^ mutation further supports the notion that hyperactivation of global RAS signaling is toxic to cells. We conclude that KRAS^Q61L^ displays elevated activation across transcriptional, proteomic, and signaling analyses, crossing a threshold that triggers both proteasomal degradation of KRAS and apoptosis as protective mechanisms in the pancreas (Fig. 5H).

## Discussion

*KRAS* ^*Q61L*^, which encodes a constitutively active mutant, is found at higher-than-expected frequencies in lung and colon cancers and is notably absent in PDAC (16, 49). To explore mechanisms for the inability of *KRAS*^*Q61L*^ to drive pancreatic tumorigenesis, we compared KRAS^G12D^, KRAS^G12R^, and KRAS^Q61L^ in a novel DOX-inducible pancreatic cell system. We demonstrated that KRAS^Q61L^ exhibits increased MAPK pathway engagement, as shown by a protein proximity assay and significantly enhanced interaction with CRAF in an in-cell BRET assay. Transcriptomic profiling revealed that KRAS^Q61L^ activates the PDAC-KRAS transcriptional signature more robustly than both KRAS^G12D^ and KRAS^G12R^. GSEA pathway analysis showed KRAS^Q61L^ drives an exaggerated but similar transcriptional response to KRAS^G12D^, likely due to the increased activated ERK1/2 nuclear localization in KRAS^Q61L^ cells. Our data suggest that KRAS^Q61L^ does not engage in fundamentally different signaling networks but rather induces heightened activation of the canonical KRAS signaling cascade. KRAS^Q61L^ paradoxically led to an increase in cell death via apoptosis and an apparent decrease in proliferation. Our findings suggest that KRAS signaling strength, not mutant-selective protein interactions, underlies the unique consequences of the KRAS^Q61L^ mutation in the pancreas.

Previous studies have highlighted RAS mutant- and isoform-selective signaling utilizing the BioID protein proximity assay and reverse-phase protein array studies (11, 16, 28–30, 50). We predicted that KRAS^Q61L^, which is rarely detected in pancreatic cancer (less than 0.1%), would have a significantly unique interactome and transcriptional profile in pancreatic cells that would define the mechanisms that limit its ability to drive PDAC in the patient. Surprisingly, the largest differences detected in the protein proximity assay were differences in the magnitude of interactions, not in the specificity of interactions. In our pancreatic model system, we detected that the dominant activated pathway was the RAF/MEK/ERK MAPK pathway, in line with recent findings (38, 39). Analysis of our transcriptional data supports this conclusion as well. Thus, although KRAS mutants may be able to interact with a diverse array of signaling pathways in bladder, melanoma, and colon models (50), our data support the conclusion that KRAS largely activates only the MAPK pathway in pancreatic cells.

An interesting observation from our TurboID analysis was the appearance of nuclear-localized proteins and the increased interaction of KRAS^Q61L^ with the nuclear export protein XPO5. Mutant KRAS has recently been described to interact with RANGAP1 to promote the activation of XPO1 independent of RAF or PI3K signaling (51). As both XPO1 and XPO5 utilize GTP-loaded RAN (52), we speculate that KRAS may similarly modulate XPO5 function via modulation of RANGAP1 activity. Consequently, RANGAP1 was detected as a non-KRAS mutant-selective interaction in our study. Furthermore, our TurboID results support KRAS interacting with many nuclear-associated proteins, suggesting a broader role for KRAS in regulating nucleocytoplasmic trafficking, an area that remains largely unexplored. The functional relevance of hits like XPO5 remains to be fully determined and will be necessary to understand the full impact of mutant RAS in disease.

Biochemists have been attempting to connect the nucleotide exchange and hydrolysis kinetics to oncogenic capacity for decades (9); however, no trend has emerged that explains how the biochemical data dictate oncogenic capacity. For example, KRAS^G12R^ and KRAS^Q61L^ have near-zero GTP hydrolysis rates, yet KRAS^G12R^ is commonly detected in PDAC and KRAS^Q61L^ incredibly rare. Biochemically, these mutants are GTP-locked in cells, which is supported by their resistance to KRAS(off) inhibitors (53). Thus, the paradox that connects biochemical rates to oncogenic potential has stymied the RAS field for decades (54).

We propose that the oncogenic potential of specific RAS mutants is dependent on the biochemical features of the mutant protein and on the ability of mutant KRAS to promote hyperactivation of the ERK/MAPK pathway. In the case of KRAS^Q61L^, its ability to hyperactivate the ERK/MAPK pathway may result from increased direct activation of CRAF. Alternatively, as mutant KRAS can promote activation of WT RAS isoforms, the elevated ERK/MAPK activity may also be driven indirectly through hyperactivation of WT RAS proteins. It is also possible that both mechanisms contribute to the heightened signaling output. By utilizing an isogenic pancreatic cell system (HPNE cells) and stable transduction of DOX-inducible KRAS mutants, we were able to maintain exquisite control of KRAS-mutant expression in this model. Our protein proximity ligation assay showed that KRAS^G12D^, KRAS^G12R^, and KRAS^Q61L^ had remarkably similar interactomes despite significantly different levels of activated ERK signaling and transcriptional output of the PKS. We previously showed that KRAS^G12R^ cannot interact with the RAS GEF SOS1 (10). Recently, we discovered that a functional consequence of the loss of the KRAS^G12R^–SOS1 interaction is a decrease in the activation levels of WT RAS isoforms and nuclear activated ERK, providing mechanistic support for the conclusion that KRAS^G12R^ has reduced oncogenic strength (37). Thus, although the biochemical data support KRAS^G12R^ as a strong oncogene due to reduced exchange and hydrolysis kinetics, this mutant behaves as a weaker than expected oncogene in pancreatic cells due to reduced WT RAS activity.

Interestingly, cells harboring KRAS^Q61H^, like KRAS^G12R^, were insensitive to the SOS1 inhibitor BI-3406 (55) and insensitive to SOS-mediated nucleotide exchange (56), suggesting that KRAS^Q61H^ also cannot activate the WT RAS isoforms. KRAS^Q61H^ has slow exchange and hydrolysis kinetics, populating it in the GTP-bound state (9). Consequently, KRAS^Q61H^ is detected in PDAC in approximately 2% of all patients, marking a second example of a biochemically “strong” oncogene that cannot activate WT RAS isoforms. We predict then that KRAS^Q61H^, which has similar intrinsic biochemical features as KRAS^Q61L^ but cannot activate WT RAS via SOS1, only marginally exceeds the tumorigenic threshold of ERK activity. Similar conclusions can be drawn with KRAS^Q61R^ although additional experimentation is necessary to validate the ability of the additional mutants to promote WT RAS activity (57).

KRAS^Q61L^ represents a mutant that is both biochemically strong and retains the ability to activate WT RAS isoforms. Previously, it was shown that HRAS^Q61L^ nucleotide exchange is modulated by SOS1 in biochemical studies (58), suggesting that KRAS^Q61L^ may promote increased WT RAS activity similar to KRAS^G12D^. Consequently, KRAS^Q61L^ promoted extreme hyperactivation of ERK levels both in the cytoplasm and nucleus, generated the strongest PKS, and was toxic to the HPNE cells. In contrast, KRAS^G12D^ has relatively higher intrinsic GTP hydrolysis rates, leading to less direct RAF activation and increased tolerability to MAPK signaling. These data are consistent with the idea that the biochemical features of each KRAS mutant, combined with the ability to activate WT RAS isoforms, possibly via SOS1, drive global ERK activity and dictate their overall oncogenic strength in the pancreas (59, 60).

Finally, we found that KRAS^Q61L^ protein expression is limited due to elevated protein degradation levels, suggesting the possibility of an intrinsic cellular mechanism to limit the expression of this hyperactive mutant. This degradation is not due to differences in thermal stability and seems to serve a protective function, as KRAS^Q61L^ expression triggers apoptotic cell death. If the precise mechanisms that target KRAS^Q61L^ for degradation can be elucidated, they may reveal actionable pathways or cellular machinery that could be leveraged to similarly destabilize other KRAS mutants in PDAC or other cancers. Together, our results demonstrate that KRAS^Q61L^ is both hyperactive and cytotoxic in the pancreatic context, providing experimental evidence for a “Goldilocks zone” of RAS signaling, in which the absence of *KRAS*^*Q61L*^ in the patient population with PDAC is not simply due to being a weaker or less transforming mutant but rather one that crosses a threshold of MAPK hyperactivation that is detrimental to pancreatic cell survival.

Although the Goldilocks model has been postulated in *KRAS*-driven cancer (19), this has not been directly shown in pancreatic cancer. Our findings on KRAS^Q61L^, combined with existing biochemical data, support a refined model of KRAS signaling in the pancreas. Given that pancreatic KRAS signaling is predominantly ERK/MAPK-driven, we propose a more nuanced “Goldilocks” model in which KRAS must signal at an optimal level to support tumorigenesis. This balance involves multiple mechanisms related to ERK/MAPK activation, including SOS sensitivity (WT RAS activation), GTP hydrolysis, and susceptibility to KRAS GDP inhibitors, reflecting increased time spent in the inactive state. In the pancreas, lower global ERK activation is associated with less aggressive PDAC, whereas excessive activation induces toxicity and growth arrest.

Our data were mainly collected in cells of pancreatic origin, and it is likely that the thresholds for ERK signaling, as well as the KRAS interactome, differ based on cell type and origin. Therefore, although our data are representative of the role of KRAS in pancreatic cancer, these trends are unlikely to hold in other cancers, specifically NRAS-mutant melanoma, in which BRAF affinity seems to play a predictive role in oncogenic strength (35). Furthermore, this conclusion is supported by patient data, as KRAS^Q61L^ is slightly enriched in patients with lung and colon cancer relative to the expected mutation frequencies in these cancers (16). Therefore, some cancer types may be more tolerant of greater levels of ERK/MAPK pathway hyperactivation, or conversely, activation of WT RAS isoforms may function as a tumor suppressor in some cellular contexts (61, 62). Why pancreatic cells cannot tolerate KRAS^Q61L^, while lung cells are more permissive, remains a fundamental question in the field. Understanding the mechanisms that dictate an upper limit on elevated MAPK signaling in the pancreas may reveal new therapeutic strategies for PDAC, including approaches that further enhance MAPK pathway activity. Our findings provide support for the concept of therapeutically hyperactivating the ERK/MAPK pathway to induce toxicity in the pancreas (63–65).

Our findings highlight the role of KRAS^Q61L^ at early stages of disease initiation rather than late-stage disease. HPNE cells have few genetic alterations and retain an untransformed phenotype, making this an ideal model for tumor initiation. Our data suggest that KRAS^Q61L^ mutants are rare in PDAC because they trigger oncogene-induced cell death. However, it is possible that metastatic PDAC harboring a KRAS^Q61L^ mutation has less dependence on KRAS activity, and therapeutic targeting of these mutants may be less effective. For example, genetic depletion of KRAS in a KRAS^Q61L^-mutant PDAC cell line (UM2) had minimal impact on proliferation in a 2D setting (16).

Investigating how different KRAS mutants affect ERK/MAPK signaling thresholds across tissue contexts will be critical for designing precision therapies that exploit therapeutic vulnerabilities associated with signaling imbalances. Future work should explore the posttranslational regulators and degradation machinery responsible for recognizing and eliminating KRAS^Q61L^ as activation of this pathway could serve as a unique therapeutic target. Ultimately, by understanding how KRAS^Q61L^ drives significant increases in oncogenic KRAS signaling to promote stress, we may unlock new approaches to therapeutically exploit *KRAS*-driven cancers.

## Supplementary Material

Supplementary Table S1 Table S1. Raw LFQ intensities from the TurboID-KRAS experiment.

Supplemental Figure S1 Figure S1. KRASQ61L displays enhanced GTP loading, consistent with constitutive activation.

Supplemental Figure S2 Figure S2. Comparative interactome analysis of KRASG12D and KRASQ61L via proximity protein labeling and mass spectrometry.

Supplemental Figure S3 Figure S3. Gene set enrichment analysis reveals greater transcriptional activation by KRASQ61L than KRASG12D.

Supplemental Figure S4 Figure S4. KRAS mutants after 3-day doxycycline treatment.

Supplemental Figure S5 Figure S5. KRAS Q61 mutations are rare across TCGA datasets, limiting available RNA-seq data for in vivo analysis.

## Data Availability

RNA-seq data analyzed in this study are available in the Gene Expression Omnibus at accession number GSE303643 or in supplemental data. Raw LFQ intensity values used for differential expression analysis of TurboID are available in Supplementary Table S1. All other raw data are available from the corresponding author upon request.
