# Supplementary figures and images for: Pancreatic Cells Are Resistant to KRASQ61L Expression due to Hyperactive ERK/MAPK Signaling and Apoptosis Induction
Source: Cancer Res Commun. 2025 Oct 22;5(10):1865–78. doi: 10.1158/2767-9764.CRC-25-0281 (PMC12541299; doi:10.1158/2767-9764.CRC-25-0281)

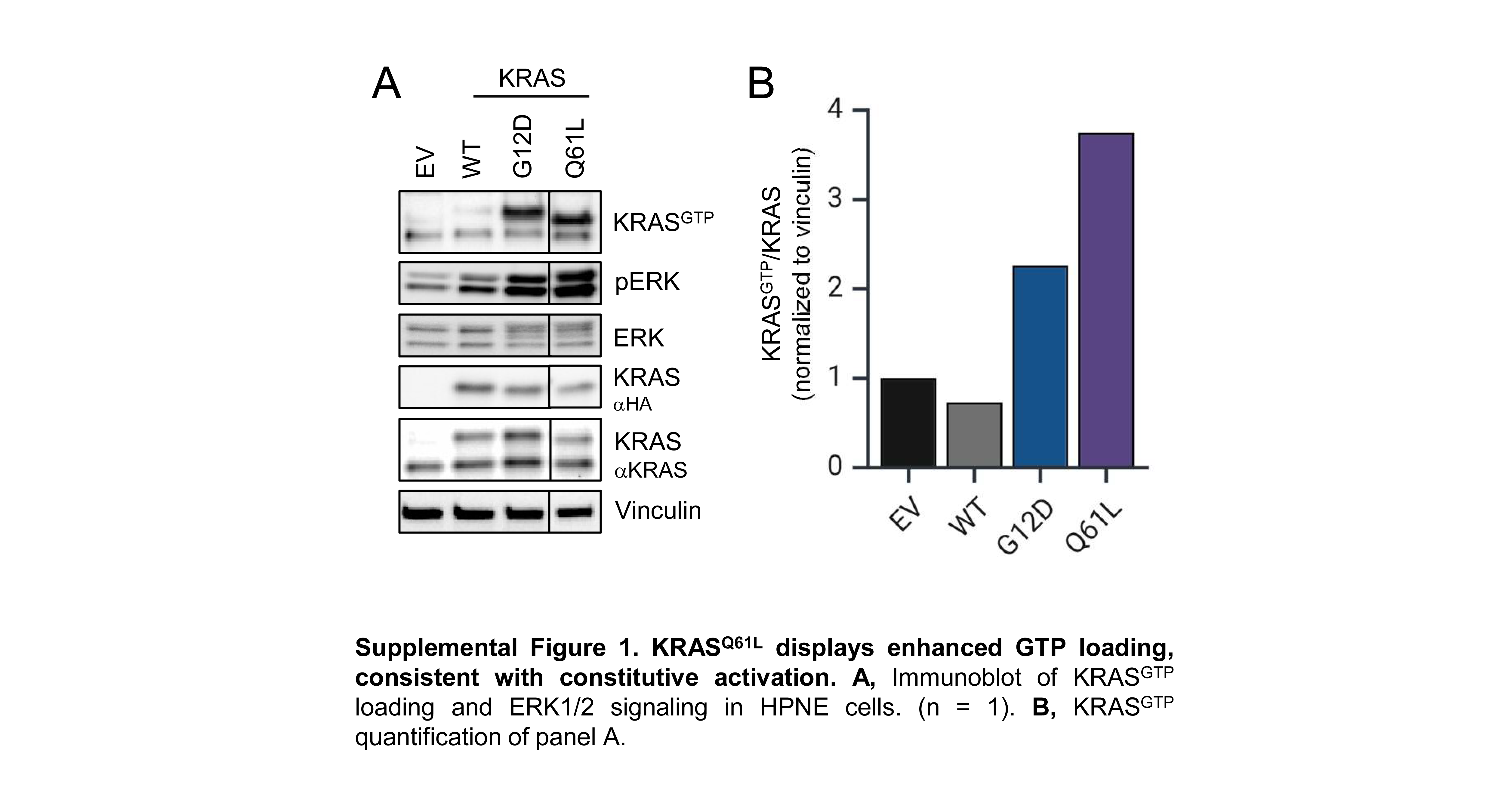

Supplement: Supplemental Figure S1 — Figure S1. KRASQ61L displays enhanced GTP loading, consistent with constitutive activation. [file crc-25-0281_supplemental_figure_s1_suppsf1.png]

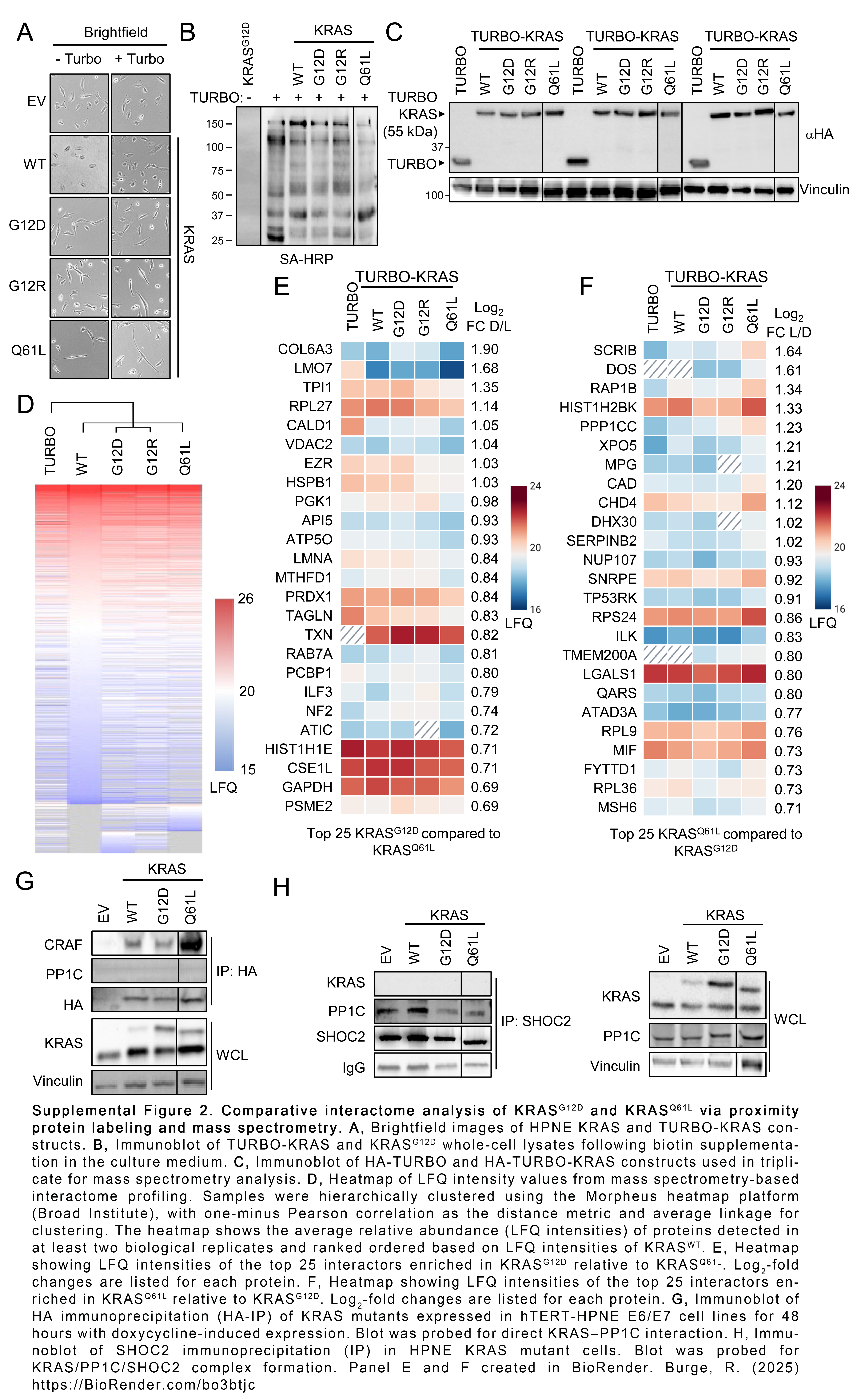

Supplement: Supplemental Figure S2 — Figure S2. Comparative interactome analysis of KRASG12D and KRASQ61L via proximity protein labeling and mass spectrometry. [file crc-25-0281_supplemental_figure_s2_suppsf2.png]

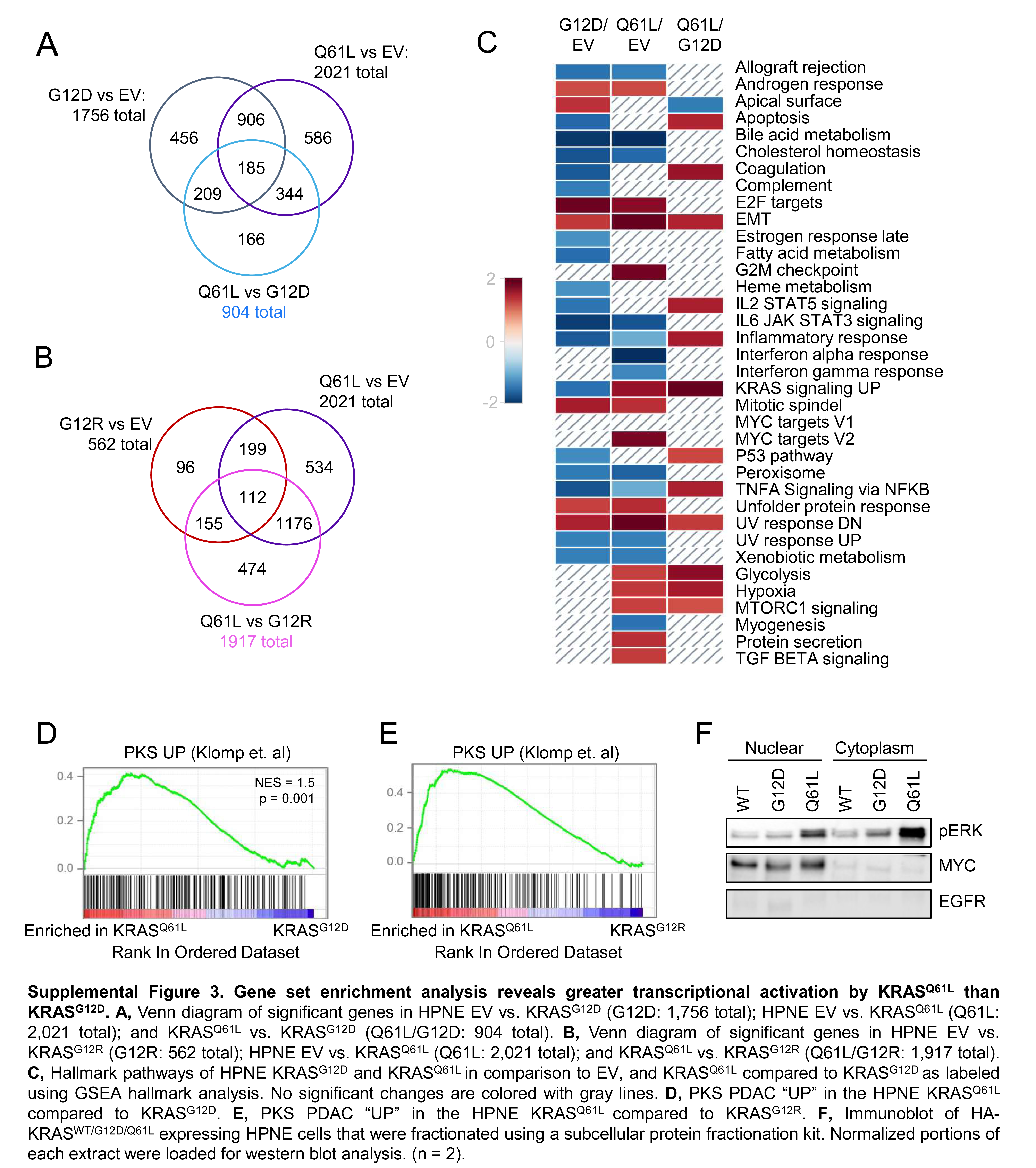

Supplement: Supplemental Figure S3 — Figure S3. Gene set enrichment analysis reveals greater transcriptional activation by KRASQ61L than KRASG12D. [file crc-25-0281_supplemental_figure_s3_suppsf3.png]

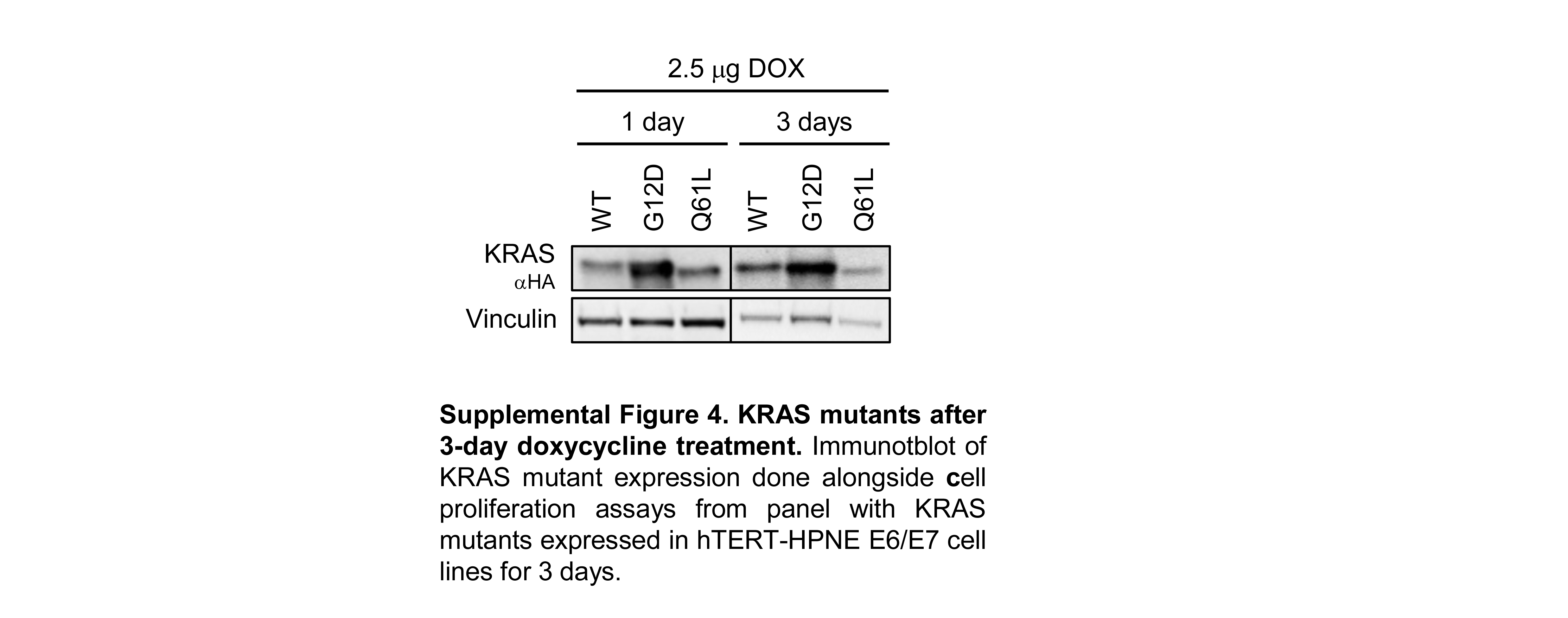

Supplement: Supplemental Figure S4 — Figure S4. KRAS mutants after 3-day doxycycline treatment. [file crc-25-0281_supplemental_figure_s4_suppsf4.png]

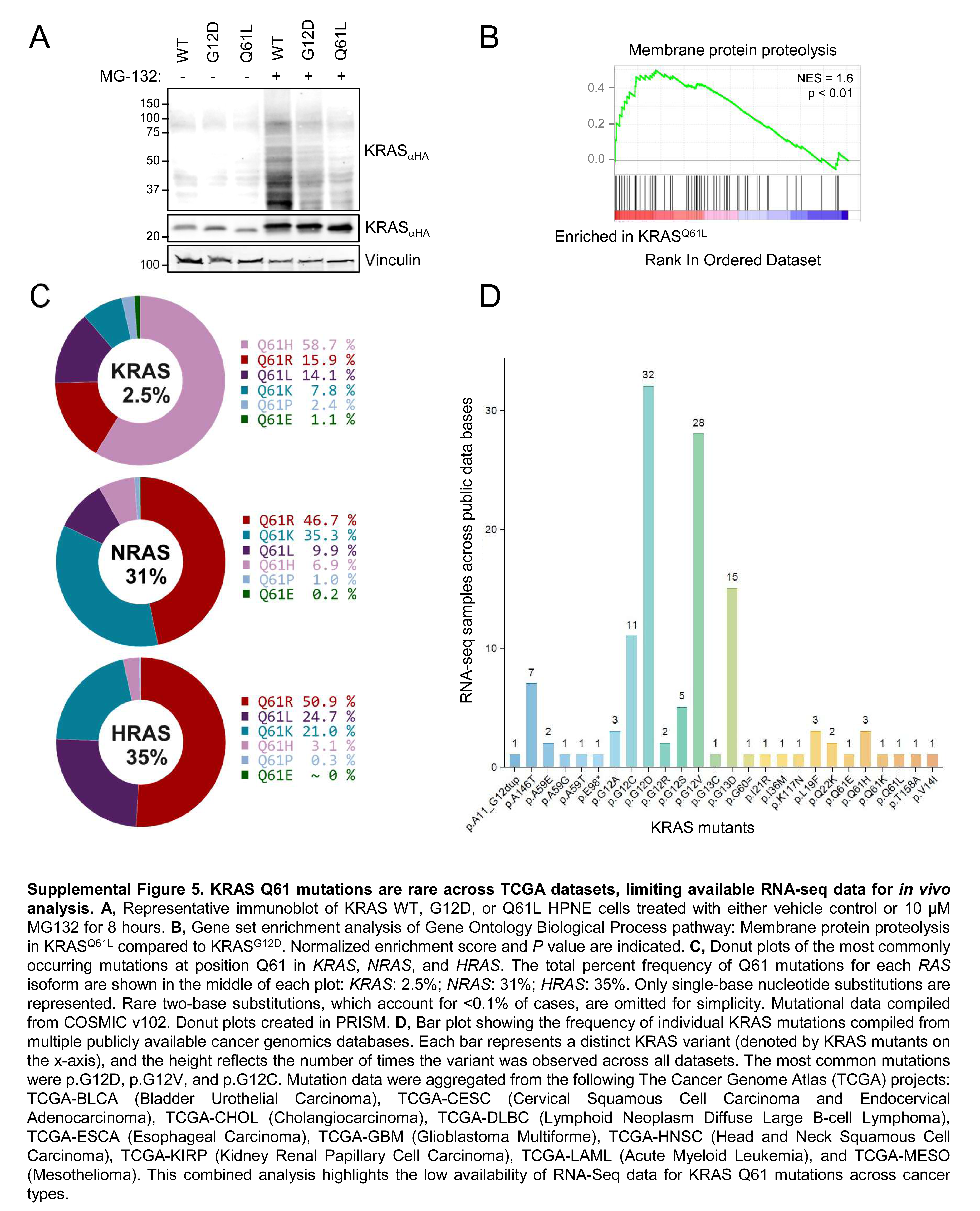

Supplement: Supplemental Figure S5 — Figure S5. KRAS Q61 mutations are rare across TCGA datasets, limiting available RNA-seq data for in vivo analysis. [file crc-25-0281_supplemental_figure_s5_suppsf5.png]
